# Two new species and one new record of *Neoperla* (Plecoptera, Perlidae) from Guangxi Zhuang Autonomous Region, China

**DOI:** 10.3897/zookeys.1053.61565

**Published:** 2021-08-23

**Authors:** Raorao Mo, Guoquan Wang, Weihai Li, Dávid Murányi

**Affiliations:** 1 Guangxi key laboratory of Agric-Environment and Agric-Products Safety and National Demonstration Center for Experimental Plant Science Education, Agricultural College, Guangxi University, Nanning, Guangxi 530004, China Guangxi University Nanning China; 2 Department of Plant Protection, Henan Institute of Science and Technology, Xinxiang, Henan 453003, China Henan Institute of Science and Technology Xinxiang China; 3 Department of Zoology, Eszterházy Károly University, Leányka u. 6, Eger H-3300, Hungary Eszterházy Károly University Eger Hungary

**Keywords:** *Neoperlamontivaga* group, Perlinae, southern China, Stoneflies, taxonomy

## Abstract

Three species of the *Neoperlamontivaga* group are described from Guangxi Zhuang Autonomous Region of southern China, including two new species, *N.falcatata***sp. nov.** and *N.shangsiensis***sp. nov.**, and a new record for Guangxi: *N.bilineata* Wu & Claassen, 1934. Illustrations and color images are provided for the three species mentioned above, and the new species is compared with related congeners in the group. Notes on the distribution of the *Neoperla* species known from Guangxi are also given.

## Introduction

*Neoperla* Needham, 1905, of the subfamily Perlinae (Plecoptera, Perlidae), is one of the most species-rich and widely distributed stonefly genera, with at least 271 species worldwide (DeWalt et al. 2020). To date, 104 *Neoperla* species have been reported in China, comprising about 38% of species in the genus (Yang and Li 2018; DeWalt et al. 2020). The *N.montivaga* species group was first proposed by Zwick (1983) and currently includes 119 species worldwide (Yang and Li 2018; DeWalt et al. 2020; Mo et al. 2020a). In the present paper, we describe two new species and provide one new record of *Neoperla* from Guangxi, southern China. The three species are members of the *Neoperlamontivaga* species group as defined by Zwick (1983), because of the incomplete sclerotization on the ventral surface of the aedeagal tube. The distribution and number of *Neoperla* species in cities of Guangxi are also presented.

## Materials and methods

Specimens were collected using an aerial net or by light traps with white lamps and stored in 75% ethanol. The holotype of the new species and other studied specimens are deposited in the Insect Collection of the Henan Institute of Science and Technology (**HIST**), Xinxiang, China and in the National Museum Prague (**NMP**), Czech Republic, as indicated in the text. Specimens were examined with the aid of a Leica M420 dissecting microscope and the color illustrations were made with a Leica S8APO. Aedeagi were everted using the cold maceration technique of Zwick (1983), and terminology follows Sivec et al. (1988). The map (Fig. 10) was prepared using a base map of the Guangxi Zhuang Autonomous Region downloaded from the standard map service of the online government service platform of the Ministry of Natural Resources, People’s Republic of China (http://bzdt.ch.mnr.gov.cn/; map number GuiS(2017)47).

## Results and discussion

### Neoperla bilineata

Taxon classificationAnimaliaPlecopteraPerlidae

Wu & Claassen, 1934

0D96EE2B-4A5C-5629-B9D8-F2647726BABC

Figs 1 , 2

Neoperla bilineata : Wu and Claassen 1934: 120; Wu 1938: 107; Claassen 1940: 158; Wu 1948: 75; Illies 1966: 268; Zwick 1973: 258; Du et al. 1999: 62; Li et al. 2017: 199; Yang and Li 2018: 39; Mo et al. 2020b: 375.

#### Material examined.

1 male (HIST), 1 male (NMP), Guangxi Zhuang Autonomous Region, Guilin City, Multinational Autonomous County of Longsheng, Jiangdi Village, terraced fields surrounded with shrubs and bamboo forest, 365m, 25°55.6'N, 110°14.8'E, 12.IV.2013, leg. M. Fikáček, J. Hájek and J. Růžička.

#### Diagnosis.

Males of this species are characterized by tergum 7 with an anteromedian pair of nipple-shaped processes, the straight aedeagal tube and the spinulose aedeagal sac with a small dorsoapical butterfly-shaped sclerite.

#### Distribution.

China (Guangxi, Sichuan).

#### Ecology.

The Jiangdi Village is located in the northeast of the Multinational Autonomous County of Longsheng, Guangxi, and is bordered by the Xing’an, Lingchuan and Ziyuan counties of Guangxi and by Chengbu County of Hunan Province. The Sangjiang River system flows through the village. In addition, both Jiangdi Village and Huaping National Nature Reserve of the same county belong to the Nanling area. The village is more than 600 km from Yibin City of Sichuan Province, where the type material of this species were collected. The adults of the species fly in spring and occur at low altitude. At the same locality, accompanying stoneflies were *Neoperladelphina* Li, Mo & Wang, 2020 and one unidentified female *Togoperla* sp.

#### Remarks.

**Figure 1.**
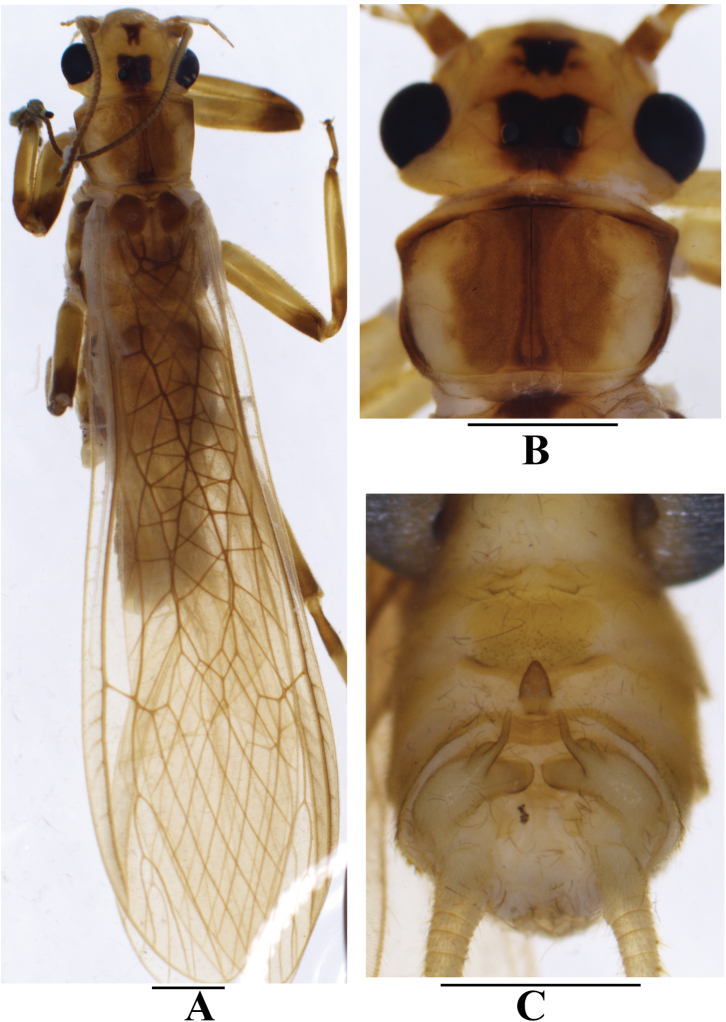
*Neoperlabilineata* Wu & Claassen, 1934 (male) **A** adult habitus, dorsal view **B** head and pronotum, dorsal view **C** terminalia, dorsal view. Scale bars: 1.0 mm.

**Figure 2.**
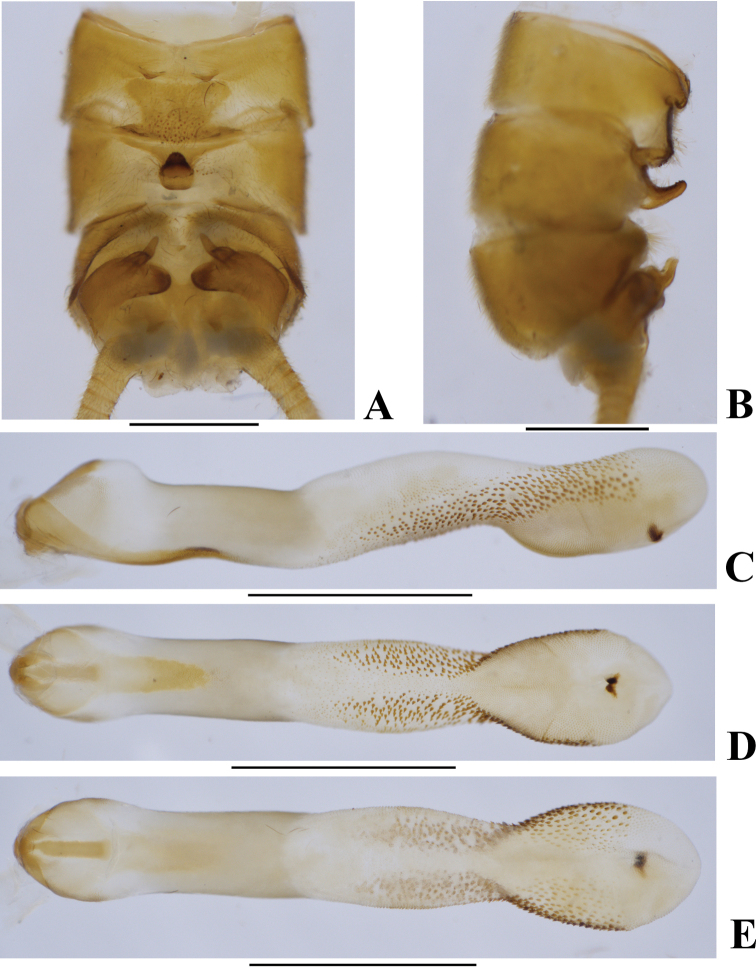
*Neoperlabilineata* Wu & Claassen, 1934 (male) **A** terminalia after being cleared, dorsal view **B** terminalia after being cleared, lateral view **C** aedeagus, lateral view **D** aedeagus, dorsal view **E** aedeagus, ventral view. Scale bars: 1.0 mm.

*Neoperlabilineata* Wu & Claassen, 1934 was originally described from the Sichuan Province of southwest China, without full eversion of the aedeagus. Li et al. (2017) made a complementary description, including that of the completely everted aedeagus. In the present study, two males of this species from Guangxi agree well with original and complementary illustrations and descriptions of the head pattern, the dorsal aspects of the terminalia and the aedeagus (Wu and Claassen 1934; Wu 1938; Li et al. 2017). We provide additional illustrations (Figs 1, 2) for aiding the recognition of this species from the Guangxi Zhuang Autonomous Region of southern China.

### Neoperla falcatata sp. nov.

Taxon classificationAnimaliaPlecopteraPerlidae

3DA6A2FB-CAA2-5C1C-83AC-AF475DAC8055

http://zoobank.org/1FD1B32C-B857-46DF-837D-0F5CF8F29A70

Figs 3 , 4 , 5 , 6

#### Type Material.

***Holotype*:** male (NMP), China: Guangxi Zhuang Autonomous Region, Shangsi County, Shiwandashan National Forest Park, forested river valley, 290–360m, 21°54.4'N, 107°54.2'E, 5–9.IV.2013, light trap, leg. M. Fikáček, J. Hájek and J. Růžička. ***Paratypes***: 2 males (NMP), 1 female and 1 male (mating pair, HIST), same data as for holotype.

#### Diagnosis.

This species is characterized by a median dark brown oval area on the head. The aedeagus is sickle-shaped, mostly covered with mixed armatures of spines and spinules. Females of this species have a small truncate tab-like subgenital plate of sternum 8.

#### Description.

**Figure 3.**
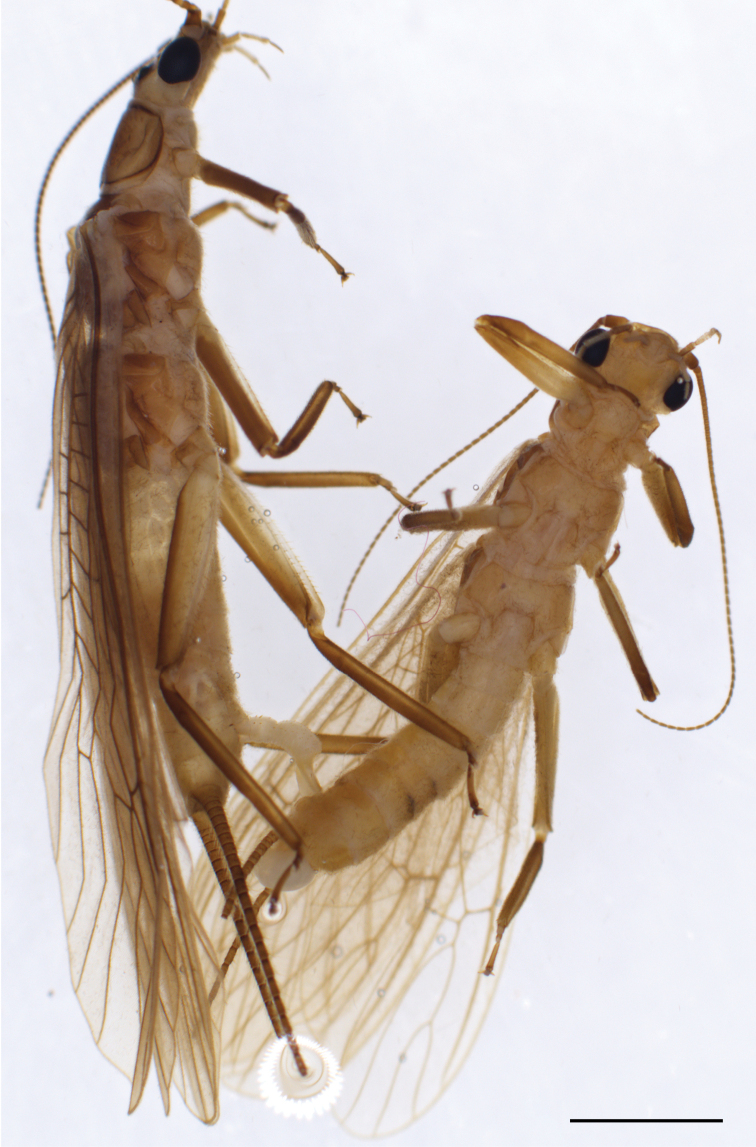
*Neoperlafalcatata* sp. nov. (mating pair). Scale bar: 2.0 mm.

**Figure 4.**
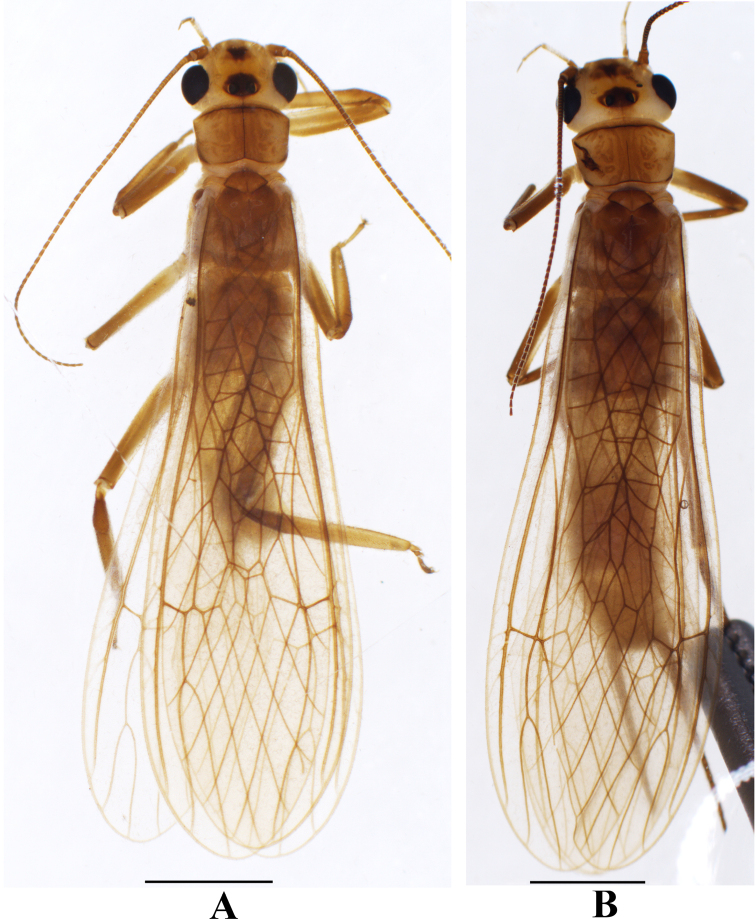
*Neoperlafalcatata* sp. nov. **A** male adult habitus, dorsal view **B** female adult habitus, dorsal view. Scale bars: 2.0 mm.

**Figure 5.**
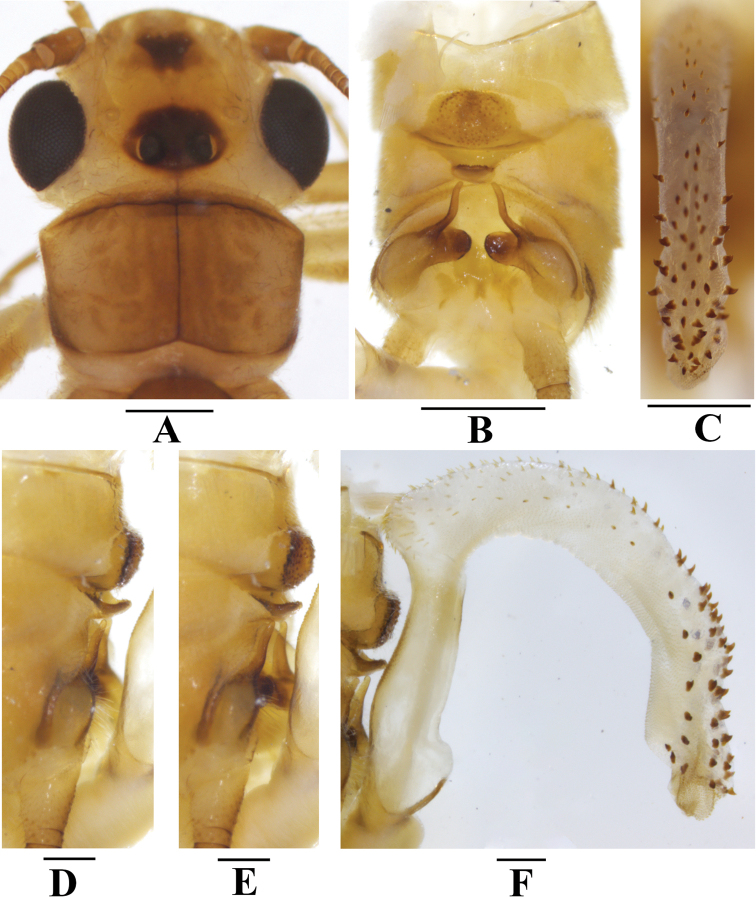
*Neoperlafalcatata* sp. nov. (male) **A** head and pronotum, dorsal view **B** terminalia after being cleared, dorsal view **C** apical half of aedeagal sac, dorsal view **D** terminalia after being cleared, lateral view **E** terminalia after being cleared, oblique lateral view **F** aedeagus, lateral view. Scale bars: 0.5 mm (**A–C**); 0.2 mm (**D–F**).

**Figure 6.**
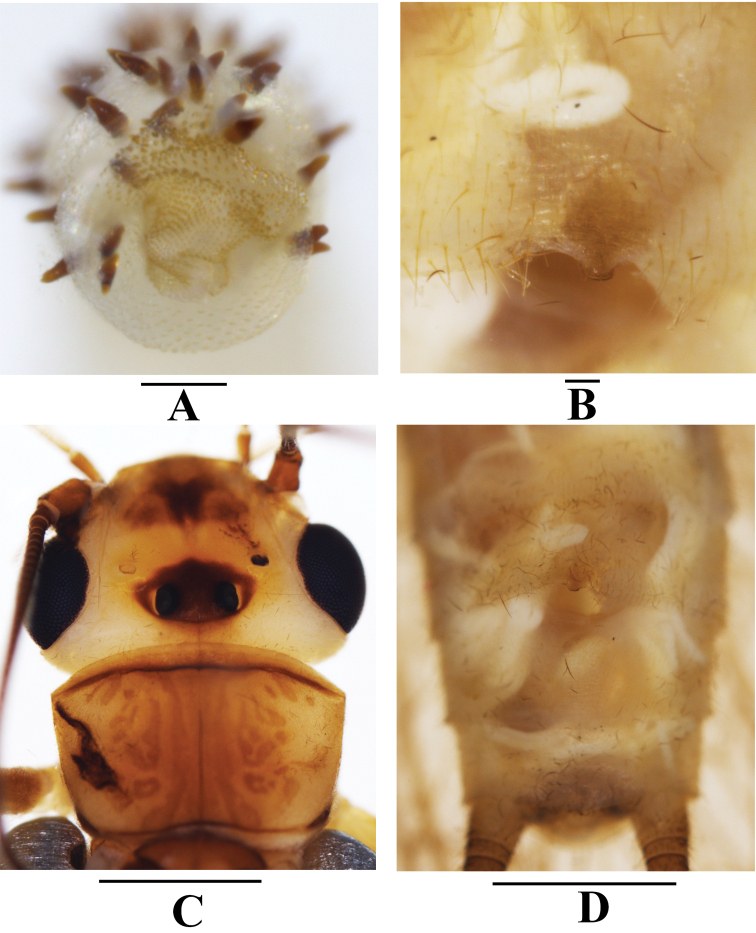
*Neoperlafalcatata* sp. nov. **A** tip of aedeagal sac, dorsal view **B** female subgenital plate, ventral view **C** female head and pronotum, dorsal view **D** female terminalia, ventral view. Scale bars: 0.1 mm (**A, B**); 1.0 mm (**C, D**).

Adult habitus (Figs 3, 4, 5A, 6C). Body color brown. Head brown, with a dark brown subtrapezoidal stigma on anterior part of frons and a distinct median dark brown oval area covering the posterior ocelli; mouthparts brown, palpi paler, antennae dark brown; biocellate, the distance between ocelli ca. 1.5× the diameter of each ocellus; head slightly wider than pronotum. Pronotum rectangular, brown, with scattered darker rugosities; anterior corners sharp, posterior corners rounded. Wings brownish and transparent, veins dark brown; legs brown except femora yellow-brown at base; cerci dark brown.

***Male*** (Figs 3, 4A, 5, 6A). Forewing length 10.0–10.5 mm. Hindwing length 8.3–8.8 mm. Posterior process of tergum 7 trapezoidal, covered with many basiconic sensilla. Tergum 8 with a tongue-shaped upcurved process, with many basiconic sensilla at apex. Tergum 9 with two paramedial patches of long hairs, without sensilla patches. Hemitergal processes of tergum 10 sclerotized, slender, finger-like, S-shaped: basally incurved, curved outward medially but incurved again apically, nearly extending to tongue-shaped process of tergum 8 (Fig. 5B, D, E). Aedeagus mostly membranous, sickle-shaped (Figs 5C, F, 6A). Aedeagal tube plump, straight, distinctly sclerotized basally and dorsally, with many spinules on dorsal surface (Fig. 5F). Aedeagal sac ca. 2.0× as long as tube, membranous, strongly curved ventrad, forming an open loop with a blunt tip; ventral surface fully armed with spinules; dorsal surface of basal half covered with brownish spines; distal half mostly covered with larger brown dorsal spines before spinous apex (Figs 5C, F, 6A).

***Female*** (Figs 3, 4B, 6B–D). Forewing length 13.8–14.5 mm. Hindwing length 12.2–12.8 mm. Generally similar to male. Posteromedial portion of sternum 8 slightly produced, forming a small, rectangular subgenital plate with a truncate tip.

#### Etymology.

The name refers to the sickle-shaped aedeagus. The Latin “falcatus” means sickle-shaped.

#### Distribution.

China (Guangxi).

#### Ecology.

Shiwandashan National Forest Park is located in the southwest of Shangsi County of Fangchenggang City, Guangxi, and it belongs to the Shiwanda Mountains. The adults of the new species fly in spring and occur at low altitude. Other accompanying stoneflies were: *Amphinemurahamiornata* Li & Yang, 2008, *Neoperlashangsiensis* sp. nov., *N.yentu* Cao & Bae, 2007, *N.yao* Stark, 1987, an unidentified *Neoperla* sp. found only as a female, *Rhopalopsolecestroidea* Li, Murányi & Gamboa, 2017, and *Togoperlaperpicta* Klapálek, 1921.

#### Remarks.

The new species shares a similar shape of the aedeagus with *Neoperlanigromarginata* Li & Zhang, 2014 from Henan Province, central China. However, the new species can be easily separated from the latter by the markings on the head and pronotum (see Li and Zhang 2014: fig. 1a). The new species can also be distinguished from *N.nigromarginata* by the hemitergal processes of tergum 10 and details of tergum 7. In *N.falcatata*, the hemitergal processes of tergum 10 are longer and nearly extend to the process of tergum 8, and tergum 7 only bears a trapezoidal posterior process, whereas in *N.nigromarginata* the hemitergal processes of tergum 10 are short and only extend to the posterior margin of tergum 9, and tergum 7 has a pair of upraised, nipple-shaped processes in addition to the distal subquadrate process. Additionally, both species can be distinguished by details of the aedeagal armature (see Li and Zhang 2014: figs 1d, 2). Additionally, the aedeagal sac of *N.nigromarginata* bears paired apical flagella, whereas in the new species the aedeagal sac lacks the apical flagella.

Finally, the female subgenital plates of the two species have different details. In *N.falcatata*, the posterior margin of the subgenital plate is truncate, while in *N.nigromarginata* the posterior margin of the subgenital plate has a slightly emarginate tip.

### Neoperla shangsiensis sp. nov.

Taxon classificationAnimaliaPlecopteraPerlidae

F6CBCA4E-904B-551B-8C30-771022DFB8BF

http://zoobank.org/89F9F7F0-D72F-49E2-A9FC-AD9C72F31DC9

Figs 7 , 8 , 9

#### Type Material.

***Holotype*:** male (NMP), China: Guangxi Zhuang Autonomous Region, Shangsi County, Shiwandashan National Forest Park, forested river valley, 290–360m, 21°54.4'N, 107°54.2'E, 5–9.IV.2013, light trap, leg. M. Fikáček, J. Hájek and J. Růžička.

#### Diagnosis.

This species is characterized by head with a median dark-brown rectangular marking covering the posterior ocelli, with a pair of posterior wing-like extensions. In the male, the aedeagus is characterized by a short ventral spinulose lobe, a pair of small dorsal spinulose lobes, and a low ventroapical lobe fully armed with spines.

#### Description.

**Figure 7.**
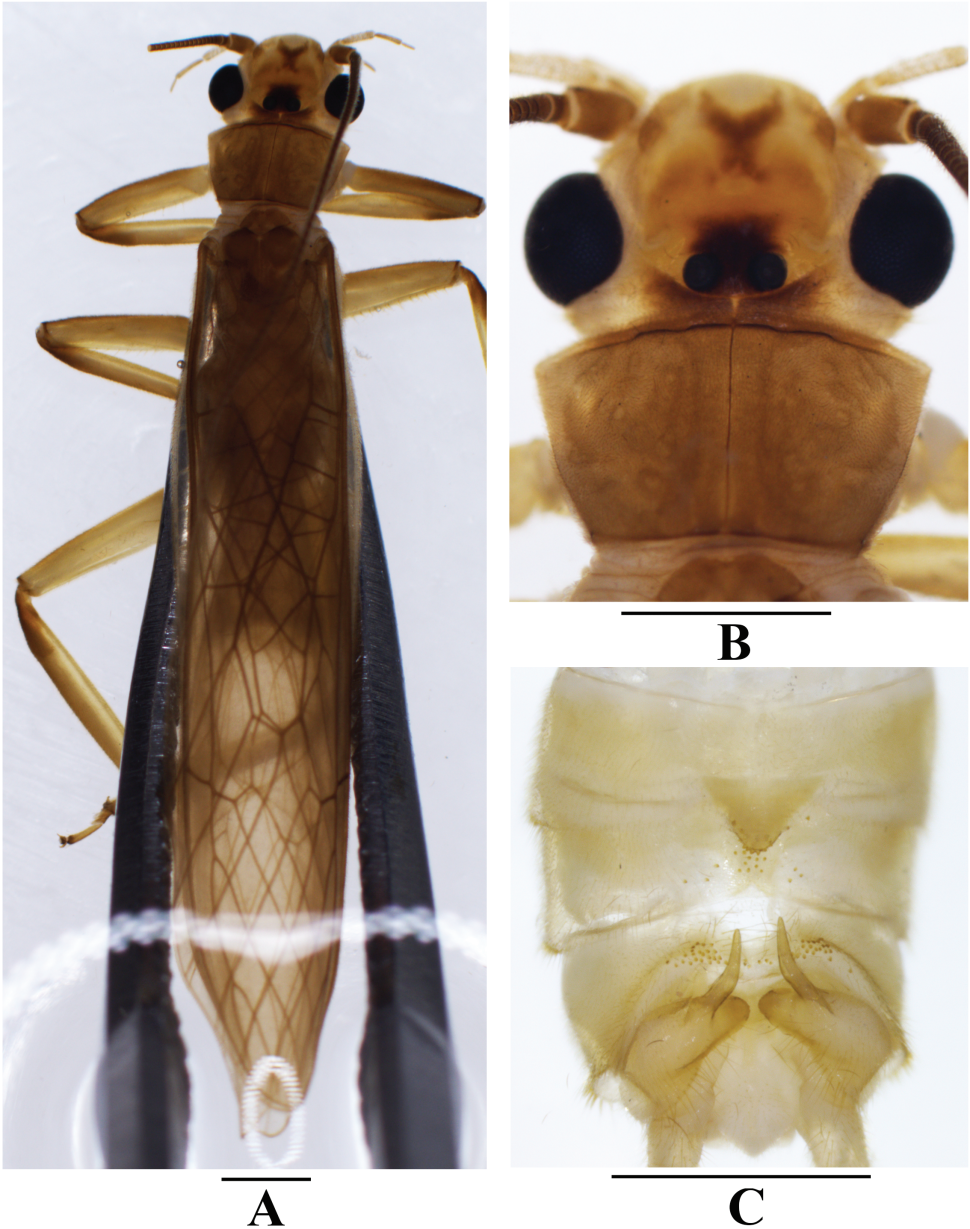
*Neoperlashangsiensis* sp. nov. (male) **A** adult habitus, dorsal view **B** head and pronotum, dorsal view **C** terminalia, dorsal view. Scale bars: 1.0 mm.

Adult habitus (Fig. 7A, B). Body color brown. Head brown, with a dark brown fishtail stigma on anterior part of frons and a distinct median dark-brown rectangular marking covering posterior ocelli, with a pair of posterior wing-like extensions; mouthparts brown, palpi paler, antennae dark brown; biocellate, the distance between ocelli slightly wider than the diameter of one ocellus; head slightly wider than pronotum. Pronotum trapezoidal, brown, with scattered darker rugosities; anterior corners sharp, posterior corners rounded. Wings brownish and transparent, veins dark brown; legs brown except femora yellow-brown at base; cerci brown.

**Figure 8.**
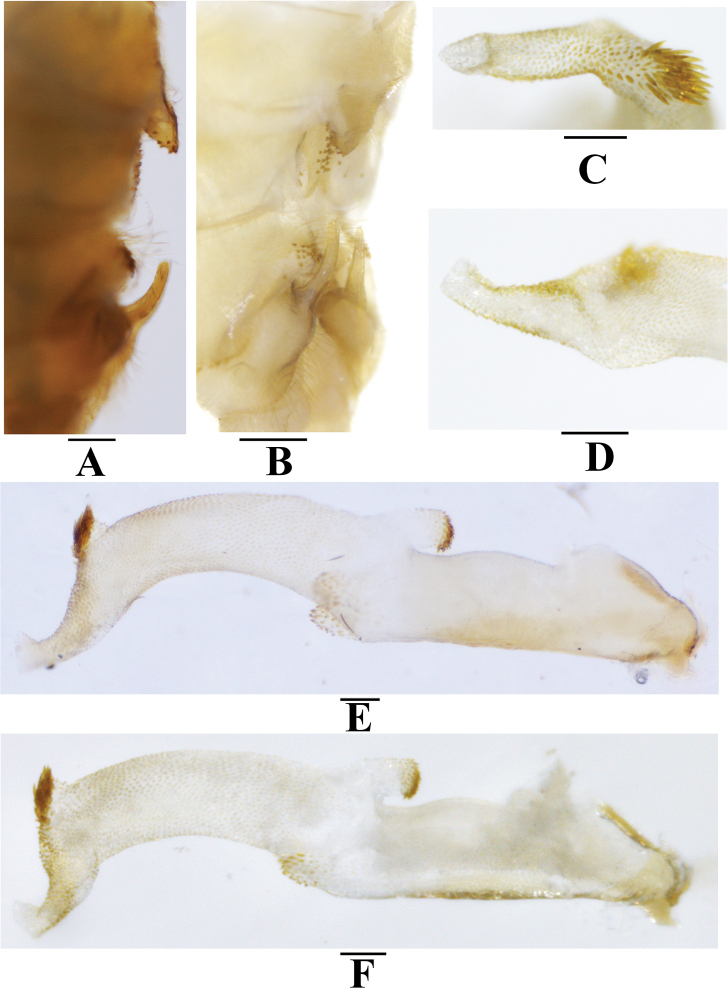
*Neoperlashangsiensis* sp. nov. (male) **A** terminalia after being cleared, lateral view **B** terminalia after being cleared, oblique lateral view **C** tip of aedeagal sac, dorsal view **D** tip of aedeagal sac, ventral view **E** aedeagus, oblique lateral view **F** aedeagus, lateral view. Scale bars: 0.2 mm (**A, B**); 0.1 mm (**C–F**).

**Figure 9.**
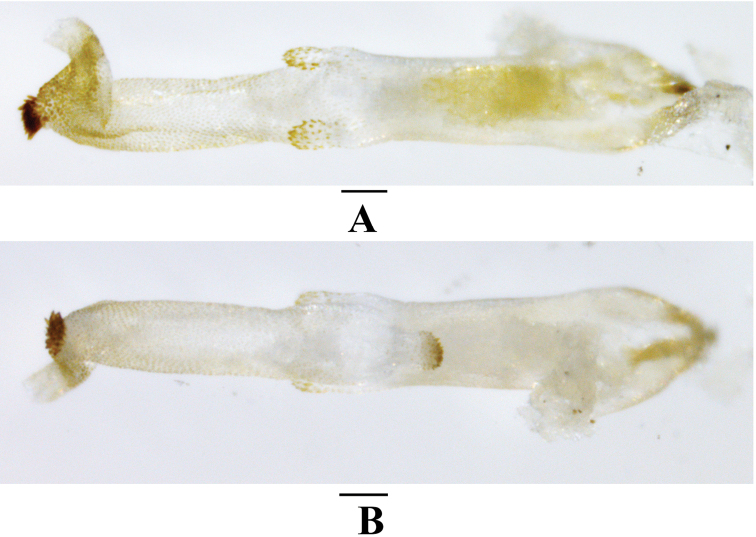
*Neoperlashangsiensis* sp. nov. (male) **A** aedeagus, dorsal view **B** aedeagus, ventral view. Scale bars: 0.1 mm.

***Male*** (Figs 7–9). Forewing length ca. 12.0 mm. Hindwing length ca. 10.4 mm. Posterior process of tergum 7 small, triangular, covered with many marginal basiconic sensilla. Tergum 8 without a process but covered by a small median spine patch. Tergum 9 with a pair of patches of basiconic sensilla and long hairs. Hemitergal processes of tergum 10 sclerotized, finger-like, bent ventrally in basal half, apical half slightly outcurved, tapering to an acute tip and nearly extending over tergum 9 (Figs 7C, 8A, B). Aedeagus mostly membranous, nearly straight (Figs 8C–F, 9). Aedeagal tube plump, straight, sclerotized basally and dorsally, with a pair of dorsoapical spinose lobes (Figs 8E, F, 9). Aedeagal sac about as long as tube, membranous, with a blunt trumpet-shaped tip; basoventral lobe distinctly shorter than corresponding width of sac, cylindrical, nearly parallel with aedeagus, with spinose apex; apical dorsal lobe small, triangular in lateral view, covered with a group of long spines; most of surface armed with dense spinules; apical portion covered with large spines, ventral spines smaller (Figs 8C–F, 9).

***Female*.** Unknown.

#### Etymology.

The species name refers to the Shangsi County, where the type locality is located.

#### Distribution.

China (Guangxi).

#### Ecology.

See ecology of *Neoperlafalcatata* sp. nov.

#### Remarks.

The new species belongs to the *Neoperlaoculata* species complex of the *montivaga* species group as defined by Zwick (1983, 1986), because of the wide lobe of tergum 7 and the four lobes of the aedeagus. The new species seems closely related to *N.furcomaculata* Kong & Li, 2016 from Hainan Province in having similar terminalia and aedeagal structures. However, the new species is easily distinguishable from the latter by the distinctively pigmented head pattern. Additionally, both species can be distinguished by the details of the aedeagal structure (see Kong et al. 2014: figs. 5d, 6). In *N.shangsiensis*, the paired dorsoapical spinose lobes of the aedeagal tube are higher and larger, and the ventroapical lobe of the aedeagal sac is distinctly enlarged. In *N.furcomaculata*, the paired dorsoapical spinose lobes of the aedeagal tube are obviously lower and smaller, and the ventroapical part of the aedeagal sac is not enlarged and is without a lobe. In addition, the basoventral lobe of aedeagal sac of the new species is nearly parallel with the aedeagal tube and distinctly shorter than the corresponding width of the sac, whereas in *N.furcomaculata* the basoventral lobe of the aedeagal sac is perpendicular to the aedeagal tube and subequal in length to the width of the corresponding portion of the sac.

## Concluding remarks

**Figure 10.**
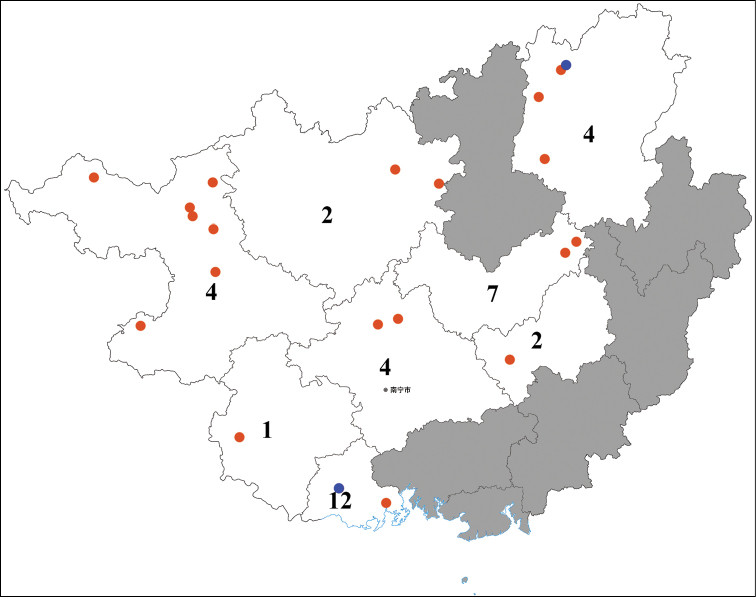
Distribution and species numbers of *Neoperla* Needham, 1905 in cities of Guangxi. Cities without records shaded in grey; blue dots indicate location of the two new species and new record.

Guangxi Zhuang Autonomous Region is located in southern China. The Region borders Guangdong Province to the east, Beibu Gulf to the south and Hainan across the sea, Yunnan Province to the west, Hunan Province to the northeast, and Guizhou Province to the northwest. The Region also borders Vietnam to the southwest. To date, 24 species of *Neoperla* known from Guangxi had been studied, mainly by Wu (1948), Yang and Yang (1990), Du (1998), Du and Sivec (2004), Li et al. (2013, 2020), Wang et al. (2013a, 2013b), Yang et al. (2017), and Mo et al. (2019, 2020a). The type locality of one of these species, *Neoperlaquadrispina* Li, Mo & Wang, 2020, was misspelled in the original description and it should be revised to Pingtianshan National Forest Park. In the checklist of Yang and Li (2018), *N.han* Stark, 1987 was recorded from Guangxi by mistake, but this species do not occur in Guangxi (Stark 1987; Du et al. 1999; Wang et al. 2013; Mo et al. 2020b). The stonefly genus *Neoperla* primarily occurs in the Baise, Fangchenggang, Guilin, Laibin and Nanning cities of Guangxi (Fig. 10). There are famous reserves or parks in these five cities, including Cenwanglaoshan National Natural Reserve, Damingshan National Natural Reserve, Dayaoshan National Nature Reserve, Huaping National Nature Reserve, Jinhuacha Nature Reserve, and Shiwandashan National Forest Park. The genus *Neoperla* is widely distributed in Guangxi, which nearly covers two-thirds of the region. It has been poorly collected in southeast Guangxi. In the present study, two additional new species of *Neoperla* are described and one new species record of the genus for Guangxi is provided.

## Supplementary Material

XML Treatment for Neoperla bilineata

XML Treatment for Neoperla falcatata

XML Treatment for Neoperla shangsiensis
